# Supplementary material for: Branching mechanism of photoswitching in an Fe(II) polypyridyl complex explained by full singlet-triplet-quintet dynamics
Source: Commun Chem. 2023 Jan 9;6:7. doi: 10.1038/s42004-022-00796-z (PMC9829715; doi:10.1038/s42004-022-00796-z)
Supplement: Supplementary file 2 — Supplementary Information [file 42004_2022_796_MOESM2_ESM.pdf]

Supplementary Information for ”Branching mechanism of an Fe(II) polypyridyl complex explained by full singlet-triplet-quintet dynamics”

Tamás Rozgonyi, György Vankó, and Mátyás Pápai*

Wigner Research Centre for Physics, P.O. Box 49, H-1525 Budapest, Hungary

Email: papai.matyas@wigner.hu

Contents

Supplementary Methods **2**

Supplementary Note 1: Diabatisation and Additional Population Dynamics **3**

Supplementary Note 2: Analysis of Diabatic Populations along Individual Trajectories **6**

Supplementary Note 3: Model Parameters **9**

Supplementary Note 4: Validation of the Utilised DFT/TD-DFT Approach **14**

Supplementary Methods

The methodology utilised in this work is based on full-dimensional trajectory surface hopping (TSH) in conjunction with a linear vibronic coupling (LVC) model.[1–3] We follow a recently developed hybrid approach:[4] while for potential energy surfaces (PESs), we use density functional theory (DFT)/time-dependent DFT (TD-DFT), we calculate spin-orbit couplings (SOCs) using multiconfigurational second-order perturbation theory (CASPT2). Importantly, this method enables the simulation of the entire singlet-triplet-quintet dynamics in full dimension with the computational bottleneck of a single CASPT2 calculation.

For details of the applied methodology, we refer to Supplementary Ref. 4; here we summarise the key aspects. Conventional on-the-fly TSH faces two problems for singlet-triplet-quintet dynamics: i) triplet-quintet SOC cannot be calculated by DFT/TD-DFT; an alternative is to use constant CASPT2 SOCs (calculated at the FC geometry), which though are not compatible with on-the-fly adiabatic PESs, and ii) the computational cost of on-the-fly electronic structure calculations is high (electronic energy, nuclear gradient), in particular for transition metal (TM) complexes with high density of states. The LVC method solves both problems as i) it is based on diabatic states, which are compatible with CASPT2 SOCs calculated at the FC geometry, and ii) is computationally very efficient. The LVC potential, which is based on the harmonic oscillator approximation and normal modes, is calculated as:

$$V^{(\alpha\alpha)} = \varepsilon^{(\alpha)} + \sum_i \kappa_i^{(\alpha)} q_i + \frac{1}{2} \sum_i \hbar\omega_i q_i^2 \tag{1}$$

$$V^{(\alpha\beta)} = \sum_i \lambda_i^{(\alpha\beta)} q_i + s^{(\alpha\beta)} \ , \tag{2}$$

Supplementary Equation 1 defines the diagonal terms; here $\varepsilon^{(\alpha)}$ is the vertical excitation energy at the FC geometry for electronic state α, q_i the normal mode coordinate for mode i, $\kappa_i^{(\alpha)}$ the linear diagonal coupling constants (forces at the FC geometry), ω_i the ground-state vibrational frequencies, and $\hbar$ the reduced Planck constant. Supplementary Equation 2 defines the off-diagonal part with $\lambda_i^{(\alpha\beta)}$ being the linear off-diagonal coupling constants (nonadiabatic couplings), and $s^{(\alpha\beta)}$ the SOC matrix elements.

The parameters of our model are given in Supplementary Note 3, Supplementary Table 1 and Supplementary Data 1.

Supplementary Note 1: Diabatisation and Additional Population Dynamics

The TSH simulations lead to electronic populations corresponding to the diagonal and adiabatic (spin-diabatic) representations, the latter being the standard basis of quantum chemistry. However, the electronic character in these bases varies as function of the nuclear geometry, which is undesirable for following electronic relaxation. We thus transformed the populations to the diabatic basis according to Supplementary Ref. 5:

$$P_{\text{diab}}^{(\alpha)}(t) = [\boldsymbol{U}^{\dagger}(t)\boldsymbol{P}_{\text{ad}}(t)\boldsymbol{U}(t)]_{\alpha\alpha} \ , \tag{3}$$

where $\boldsymbol{P}_{\text{ad}}$ and $\boldsymbol{P}_{\text{diab}}$ are the adiabatic (spin-diabatic) and diabatic population matrices, respectively, and $\boldsymbol{U}$ is the transformation matrix that diagonalises the diabatic potential matrix to yield the adiabatic one:

$$\boldsymbol{V}_{\text{ad}} = \boldsymbol{U}^{\dagger}\boldsymbol{V}_{\text{diab}}\boldsymbol{U} \ , \tag{4}$$

with the diabatic potential matrix $\boldsymbol{V}_{\text{diab}} = \boldsymbol{V}$ defined by Supplementary Equations 1 and 2. This diabatisation procedure leads to the populations shown in Figures 2 and 3 of the main article, with the populations corresponding to the same spin multiplicity and electronic character summed up (^{1}MLCT, ^{1}MC, ^{3}MLCT, ^{3}MC, ^{5}MC). We note that the Franck-Condon (FC) reference states can clearly be classified as MLCT or MC, based on the dominant electronic character. The largest TD-DFT MLCT/MC mixing weight is ca. 10% with significantly smaller mixing weights, in most cases; this demonstrates that the MLCT/MC mixing at the FC geometry is negligible.

As this work focuses on the excited-state dynamics leading to conversion into the quintet HS state, in Figures 2 and 3 of the main article, we show only the excited-state populations (normalised to unity). In Supplementary Figure 1 below, for the full simulation, we include the ground-state ^{1}GS populations and normalise the ground-state plus the excited-state populations to unity. As seen in the figure, ^{1}GS appears as a minor component ($\sim$10% at the final time step of the simulation, 1.5 ps). For the simulation, in which the ^{3}MC states are excluded (Figure 2b of the main article), the ^{1}GS population remains zero throughout, which shows that transfer back to the ground state can only occur via the ^{3}MCs.

In Supplementary Figure 2 below, we extend Figure 3 of the main article, i.e., the excited-state population dynamics with the ^{3}MC population decomposed to the the ^{3}T$_{1g}$ and ^{3}T$_{2g}$ components, to the full simulation time 0−1500 fs.

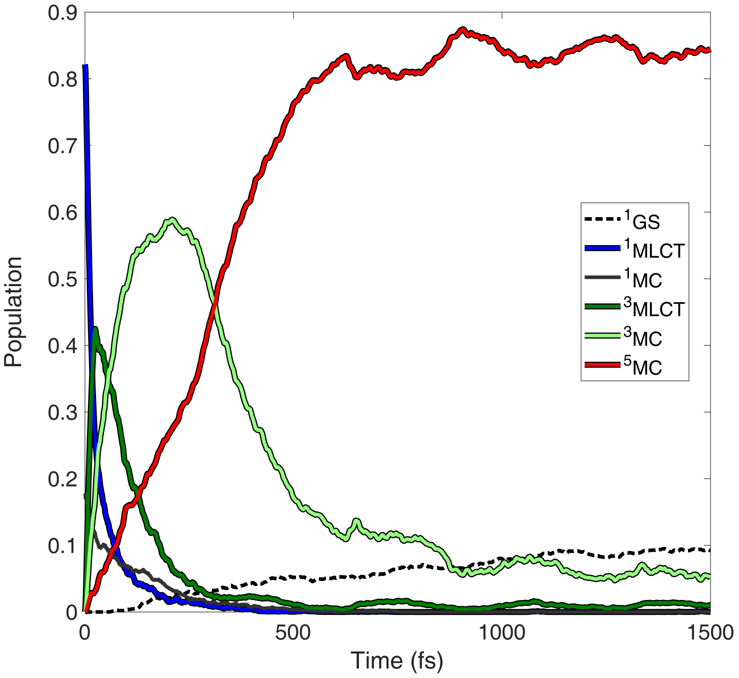

Supplementary Figure 1: Simulated excited-state population dynamics of $[Fe(terpy)_2]^{2+}$. The whole population, i.e., ground-state plus excited-state, is normalised to unity.

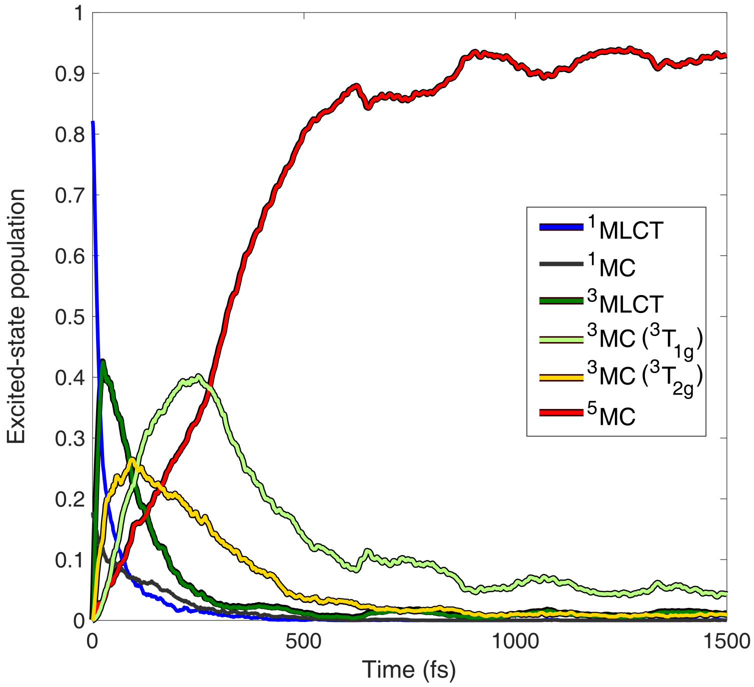

Supplementary Figure 2: Simulated excited-state population dynamics of $[Fe(terpy)_2]^{2+}$. The excited-state population is normalised to unity.

Supplementary Note 2: Analysis of Diabatic Populations along Individual Trajectories

In order to investigate which pathways build up the branching mechanism observed in Figure 3 of the main article, we analysed the diabatic populations of a representative set of 100 trajectories. In most cases, we found that the quintet state is populated via a process that involves both ^{3}MC components: ^{3}MLCT → ^{3}MC(^{3}T$_{2g}$) → ^{3}MC(^{3}T$_{1g}$) → ^{5}MC; an example of such a trajectory that clearly follows this pathway is shown in Supplementary Figure 3. We have noticed, however, that in a smaller but still considerable number of trajectories (∼10%) the situation is different: the quintet state is populated faster via a ^{3}MLCT → ^{3}MC(^{3}T$_{2g}$) → ^{5}MC process; example for this is shown in Supplementary Figure 4. Finally, we mention that we also found a single example for the third possible pathway, ^{3}MLCT → ^{3}MC(^{3}T$_{1g}$) → ^{5}MC (Supplementary Figure 5), but its weight, i.e., occurence is so low that it is negligible.

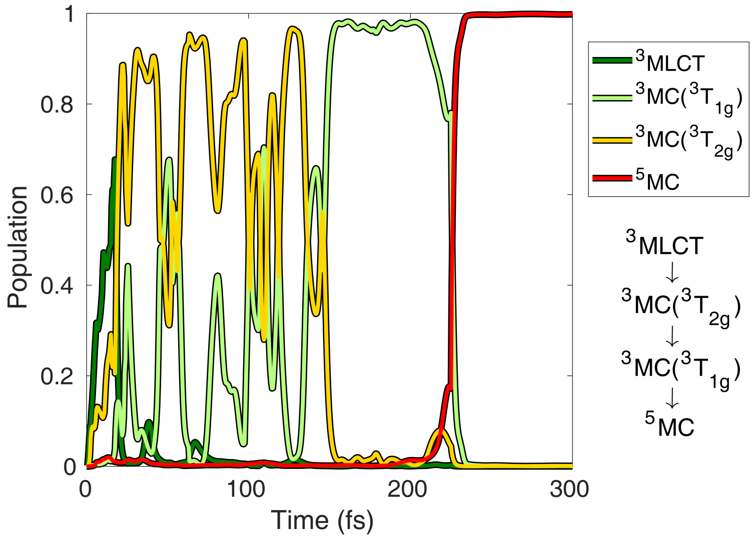

Supplementary Figure 3: Example of a trajectory for the dominant ^{3}MLCT → ^{3}MC(^{3}T$_{2g}$) → ^{3}MC(^{3}T$_{1g}$) → ^{5}MC pathway.

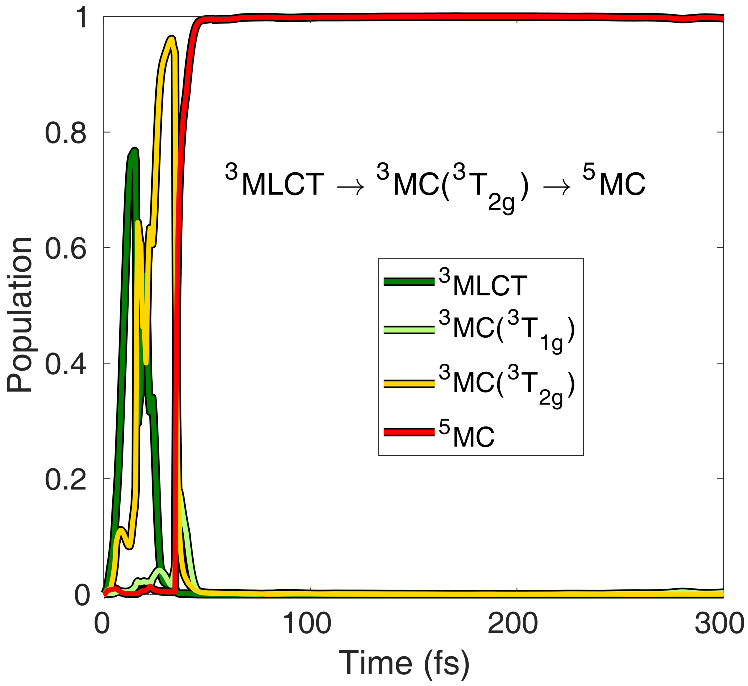

Supplementary Figure 4: Example of a trajectory for the ^{3}MLCT → ^{3}MC(^{3}T$_{2g}$) → ^{5}MC pathway.

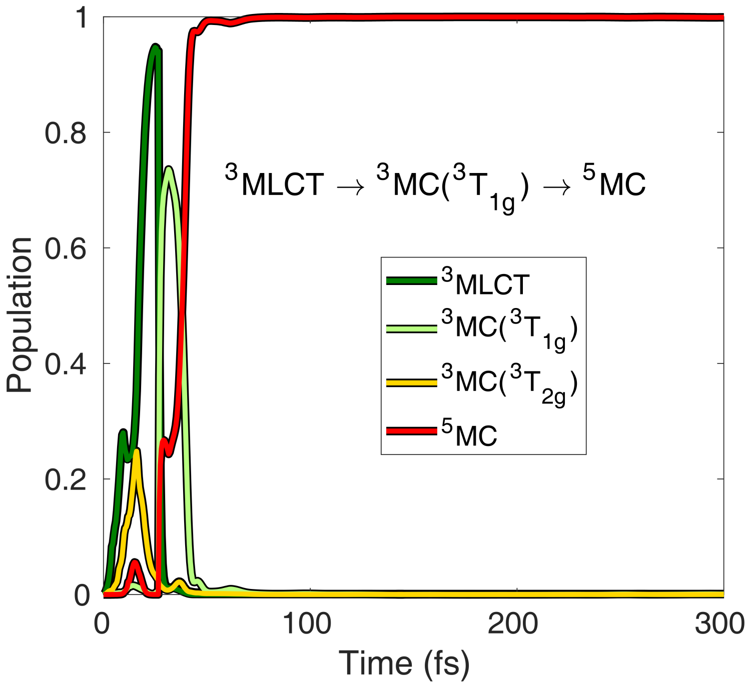

Supplementary Figure 5: Example of a trajectory for the ^{3}MLCT → ^{3}MC(^{3}T$_{1g}$) → ^{5}MC pathway.

Supplementary Note 3: Model Parameters

The ground-state normal mode frequencies are given in the data file "normal_modes_freq.dat" within Supplementary Data 1, both in eV and cm^{-1}. The frequencies of the Fe-N breathing normal mode ν_8 (where the indexing corresponds to increasing frequency, i.e., ν_1 is the lowest-frequency mode) and the antisymmetric Fe-N stretching mode ν_{12}, which are used in Figure 1 of the main article, are 104.45 cm^{-1} (0.013 eV) and 147.34 cm^{-1} (0.018 eV), respectively. The FC geometry is given in Cartesian coordinates (Å) in the data file "FC.xyz".

The $\varepsilon^{(\alpha)}$ energies, as well as the state characters, the C_2 state symmetries and oscillator strengths (for singlet excited states) of the DFT/TD-DFT electronic states (calculated at the FC geometry) are presented in Supplementary Table 1. The $\varepsilon^{(\alpha)}$ (eV) values are also provided in the data file "epsilon.dat".

The linear coefficients $\kappa_i^{(\alpha)}$ and $\lambda_i^{(\alpha\beta)}$ are given in the data files "kappa.dat" and "lambda.dat", respectively (both in eV). The SOC matrix is given in the data file "SOC.dat" (in cm^{-1}) with state ordering as defined in Supplementary Table 1; for triplet and quintet spin components, we use the following ordering: T_1 ($m_S = -1$), T_2 ($m_S = -1$), ..., T_{13} ($m_S = -1$), T_1 ($m_S = -0$), T_2 ($m_S = -0$), ..., T_{13} ($m_S = 0$), T_1 ($m_S = +1$), T_2 ($m_S = +1$), ..., T_{13} ($m_S = +1$), and similarly for the quintets Q_1 ($m_S = -2$), Q_1 ($m_S = -1$), Q_1 ($m_S = 0$), Q_1 ($m_S = +1$), Q_1 ($m_S = +2$), Q_2 ($m_S = -2$), ..., Q_2 ($m_S = +2$), Q_3 ($m_S = -2$), ..., Q_3 ($m_S = +2$).

The character of the DFT/TD-DFT and CASPT2 electronic states was checked for consistency, by analysis of the dominant electronic configurations and orbitals involved in the generation of excited states. As the composition of ligand orbitals are not that straightforward as those of Fe-3*d*-based orbitals (many more combinations are possible), below we present the L_1–L_4(terpy-π^*) ligand orbitals, both for DFT and CASSCF/CASPT2 (Supplementary Figures 6 and 7, respectively). Comparison of these two figures show that the DFT and state-averaged active orbitals agree very well.

Supplementary Table 1: C_2 state symmetries, $\varepsilon^{(\alpha)}$ energies, and oscillator strengths for excitation from the ground state (for singlet states) of the DFT/TD-DFT electronic states calculated at the FC geometry. The ground-state electronic configuration is $3d_{xz}^2 3d_{yz}^2 3d_{xy}^2 3d_{x^2-y^2}^0 3d_{z^2}^0 \mathrm{L}_1(\text{terpy-}\pi^*)^0 \mathrm{L}_2(\text{terpy-}\pi^*)^0 \mathrm{L}_3(\text{terpy-}\pi^*)^0 \mathrm{L}_4(\text{terpy-}\pi^*)^0$.

State	Character	C_2 symmetry	$\varepsilon^{(\alpha)}$ (eV)	Osc. strength
S_0 (^{1}GS)	Ground state	A	0.000	–
S_1 (^{1}MC)	$3d_{xy} \to 3d_{x^2-y^2}$	A	2.155	0.000
S_2 (^{1}MLCT)	$3d_{xy} \to \mathrm{L}_1(\text{terpy-}\pi^*)$	B	2.358	0.009
S_3 (^{1}MLCT)	$3d_{xy} \to \mathrm{L}_2(\text{terpy-}\pi^*)$	B	2.358	0.009
S_4 (^{1}MLCT)	$3d_{xz,yz} \to \mathrm{L}_{1,2}(\text{terpy-}\pi^*)$	A	2.442	0.000
S_5 (^{1}MLCT)	$3d_{xz,yz} \to \mathrm{L}_{1,2}(\text{terpy-}\pi^*)$	A	2.464	0.000
S_6 (^{1}MC)	$3d_{yz} \to 3d_{x^2-y^2,z^2}$	B	2.550	0.000
S_7 (^{1}MC)	$3d_{xz} \to 3d_{x^2-y^2,z^2}$	B	2.550	0.000
T_1 (^{3}MC)	$3d_{xy} \to 3d_{x^2-y^2}$	A	1.471	–
T_2 (^{3}MC)	$3d_{xz,yz} \to 3d_{x^2-y^2}$	B	1.699	–
T_3 (^{3}MC)	$3d_{xz,yz} \to 3d_{x^2-y^2}$	B	1.699	–
T_4 (^{3}MC)	$3d_{xz} \to 3d_{z^2}$	B	2.081	–
T_5 (^{3}MC)	$3d_{yz} \to 3d_{z^2}$	B	2.081	–
T_6 (^{3}MC)	$3d_{xy} \to 3d_{z^2}$	A	2.136	–
T_7 (^{3}MLCT)	$3d_{xz,yz} \to \mathrm{L}_{1,2}(\text{terpy-}\pi^*)$	A	2.177	–
T_8 (^{3}MLCT)	$3d_{xz,yz} \to \mathrm{L}_{1,2}(\text{terpy-}\pi^*)$	A	2.248	–
T_9 (^{3}MLCT)	$3d_{xy} \to \mathrm{L}_1(\text{terpy-}\pi^*)$	B	2.261	–
T_{10} (^{3}MLCT)	$3d_{xy} \to \mathrm{L}_2(\text{terpy-}\pi^*)$	B	2.261	–
T_{11} (^{3}MLCT)	$3d_{xz,yz} \to \mathrm{L}_{1,2}(\text{terpy-}\pi^*)$	A	2.349	–
T_{12} (^{3}MLCT)	$3d_{xz,yz} \to \mathrm{L}_{1,2}(\text{terpy-}\pi^*)$	A	2.430	–
T_{13} (^{3}MLCT)	$3d_{xy} \to \mathrm{L}_4(\text{terpy-}\pi^*)$	A	2.508	–
Q_1 (^{5}MC)	$3d_{xy}, 3d_{xz} \to 3d_{x^2-y2}, 3d_{z^2}$	B	1.927	–
Q_2 (^{5}MC)	$3d_{xy}, 3d_{yz} \to 3d_{x^2-y2}, 3d_{z^2}$	B	1.927	–
Q_3 (^{5}MC)	$3d_{xz}, 3d_{yz} \to 3d_{x^2-y2}, 3d_{z^2}$	A	1.935	–

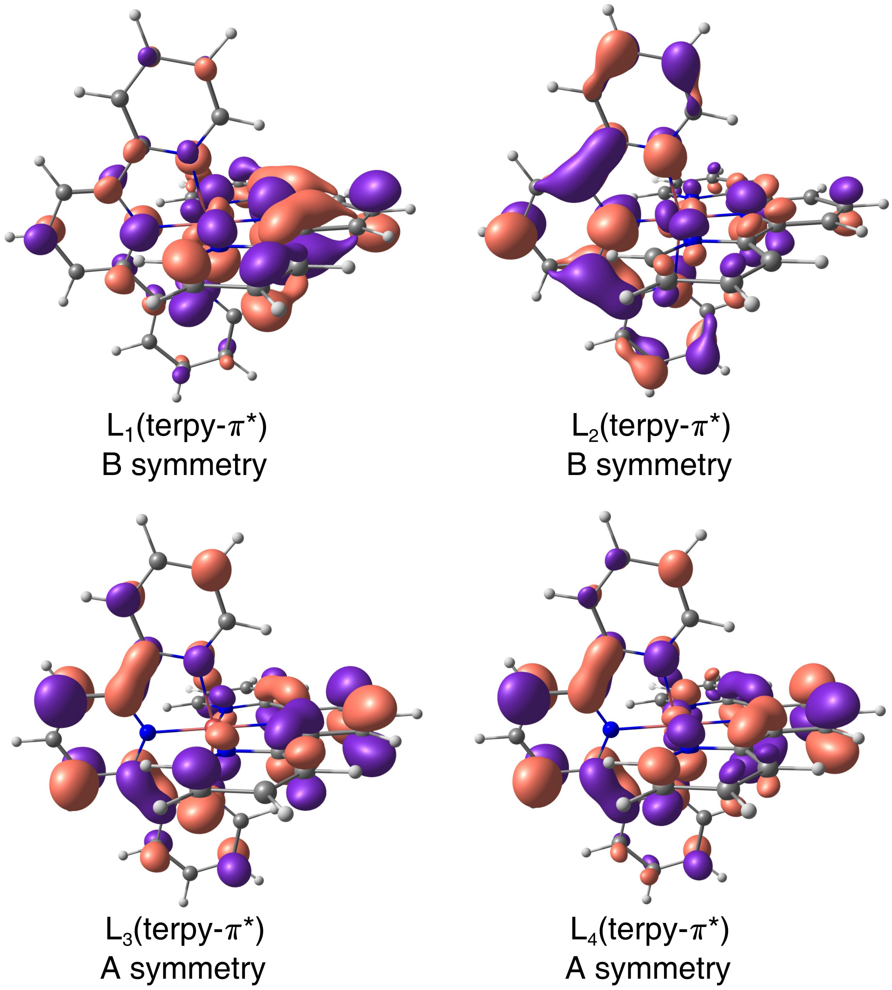

Supplementary Figure 6: DFT L_1–L_4(terpy-π^*) ligand orbitals of $[Fe(terpy)_2]^{2+}$, calculated at the ground-state equilibrium geometry.

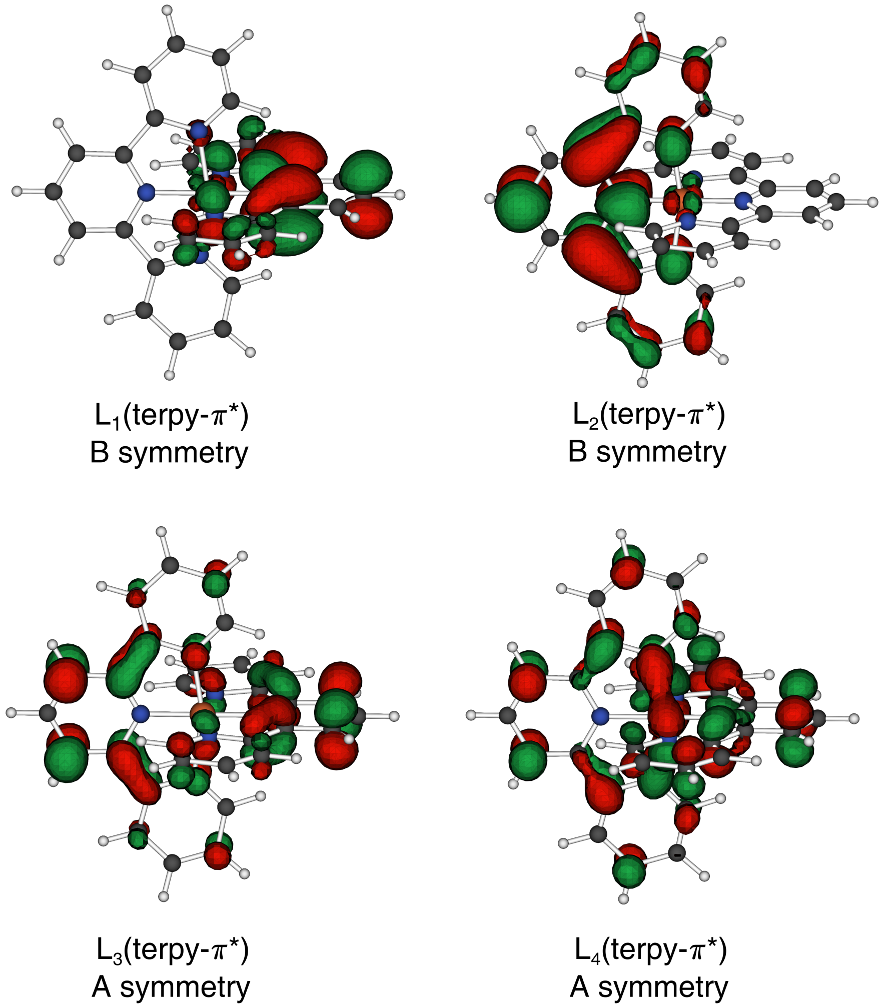

Supplementary Figure 7: State-averaged active L_1–L_4(terpy-π^*) ligand orbitals of $[Fe(terpy)_2]^{2+}$, calculated at the ground-state equilibrium geometry.

In addition to the above qualitative analysis, we assessed quantitatively the similarity of the DFT/TD-DFT vs CASPT2 states by comparison of the corresponding SOCs, which are the only parameters of our model in which the CASPT2 states enter. We note that the CASPT2 ground-state minimum is shifted significantly towards lower Fe-N bond lengths[6] (but surely other coordinates are also affected to some extent), thus the DFT/TD-DFT and CASPT2 states and SOCs were calculated at different geometries. In fact, this is the reason why we decided to compare the SOCs for which the geometry dependence is assumed to be small and neglected anyway in the LVC models, rather than directly quantify the similarity of the states by wave function overlaps, which would be misleading, as calculated for DFT/TD-DFT and CASPT2 states corresponding to different geometries. For singlet-triplet and triplet-triplet SOCs, we compare the CASPT2 and TD-DFT SOCs calculated at the ground-state equilibrium geometry. Triplet-quintet SOCs cannot

be calculated within our DFT/TD-DFT approach, as restricted Kohn-Sham (RKS) TD-DFT triplets are not compatible with unrestricted Kohn-Sham (UKS) quintets. For these triplet-quintet SOCs, we compare our CASPT2 SOCs for $[Fe(terpy)_2]^{2+}$ to the CASPT2 SOCs for $[Fe(bipy)_3]^{2+}$, taken from Sousa et al.;[7] this work also reports singlet-triplet SOCs which we also use in our comparative analysis. In the table below, the largest two SOC values are presented in cm^{-1}– with the real and imaginary parts combined into the absolute value – for each multiplicity and character. (For $[Fe(bipy)_3]^{2+}$, the largest value is given, as only this was reported in Supplementary Ref. 7. Furthermore, SOCs between triplet states were also not reported in Supplementary Ref. 7).

Supplementary Table 2: Comparison of the largest TD-DFT and CASPT2 SOCs for $[Fe(terpy)_2]^{2+}$ (this work) and $[Fe(bipy)_3]^{2+}$ (from Supplementary Ref. 7). Calculated SOC values are given in cm^{-1}.

SOC term	$[Fe(terpy)_2]^{2+}$ – CASPT2	$[Fe(terpy)_2]^{2+}$ – TD-DFT	$[Fe(bipy)_3]^{2+}$ – CASPT2
$\lvert\langle ^1MC\lvert H_{SOC}\rvert ^3MC(^3T_{1g})\rangle\rvert$	130.2, 67.4	122.2, 71.7	75.5
$\lvert\langle ^1MC\lvert H_{SOC}\rvert ^3MC(^3T_{2g})\rangle\rvert$	163.4, 137.3	156.1, 115.4	131.4
$\lvert\langle ^1MC\lvert H_{SOC}\rvert ^3MLCT\rangle\rvert$	178.2, 72.6	90.5, 73.8	164.7
$\lvert\langle ^1MLCT\lvert H_{SOC}\rvert ^3MC(^3T_{1g})\rangle\rvert$	100.5, 32.0	75.7, 48.7	96.0
$\lvert\langle ^1MLCT\lvert H_{SOC}\rvert ^3MC(^3T_{2g})\rangle\rvert$	149.9, 145.4	86.4, 57.4	214.3
$\lvert\langle ^1MLCT\lvert H_{SOC}\rvert ^3MLCT\rangle\rvert$	175.8, 175.0	180.6, 170.5	199.9
$\lvert\langle ^3MLCT\lvert H_{SOC}\rvert ^3MC(^3T_{1g})\rangle\rvert$	83.3, 51.5	46.7, 42.6	–
$\lvert\langle ^3MLCT\lvert H_{SOC}\rvert ^3MC(^3T_{2g})\rangle\rvert$	119.3, 104.6	63.8, 51.8	–
$\lvert\langle ^3MC(^3T_{1g})\lvert H_{SOC}\rvert ^3MC(^3T_{2g})\rangle\rvert$	170.9, 93.5	71.2, 41.0	–
$\lvert\langle ^3MLCT\lvert H_{SOC}\rvert ^5MC\rangle\rvert$	18.3, 12.5	–	6.2
$\lvert\langle ^3MC(^3T_{1g})\lvert H_{SOC}\rvert ^5MC\rangle\rvert$	373.1, 355.5	–	417.7
$\lvert\langle ^3MC(^3T_{2g})\lvert H_{SOC}\rvert ^5MC\rangle\rvert$	263.8, 262.7	–	219.9

While we see some systematic underestimation of the SOCs by the TD-DFT compared to the CASPT2 values, the agreement between the respective values are satisfactory and thus justifies the combination of DFT/TD-DFT PESs and CASPT2 SOCs for the studied $[Fe(terpy)_2]^{2+}$ complex. In addition, when we compare the SOCs obtained by the same CASPT2 method for the two polypyridine complexes, $[Fe(terpy)_2]^{2+}$ and $[Fe(bipy)_3]^{2+}$, we also find a good agreement, which is consistent with the close similarity in the photophysical behaviour of the two molecules.

Supplementary Note 4: Validation of the Utilised DFT/TD-DFT Approach

As in this work, we calculated excited states using two different computational approaches, namely, RKS/TD-DFT for singlet/triplet states and UKS for quintets, we carried out an additional study to validate this combination of DFT/TD-DFT methods. In Supplementary Figure 8 below, we present the PESs of $[Fe(terpy)_2]^{2+}$ along a combined coordinate that connects the LS ($^1A_{1g}$) and HS ($^5T_{2g}$) minima, calculated by the protocol utilised in the present work (RKS/TD-DFT singlet-triplet + UKS quintet), benchmarked against reference CASPT2 PESs (taken from our previous work[6]). As is clear from the figure, these results demonstrate a reasonably good qualitative agreement, with one of the most significant differences being the quintet overstabilisation by UKS with respect to CASPT2.

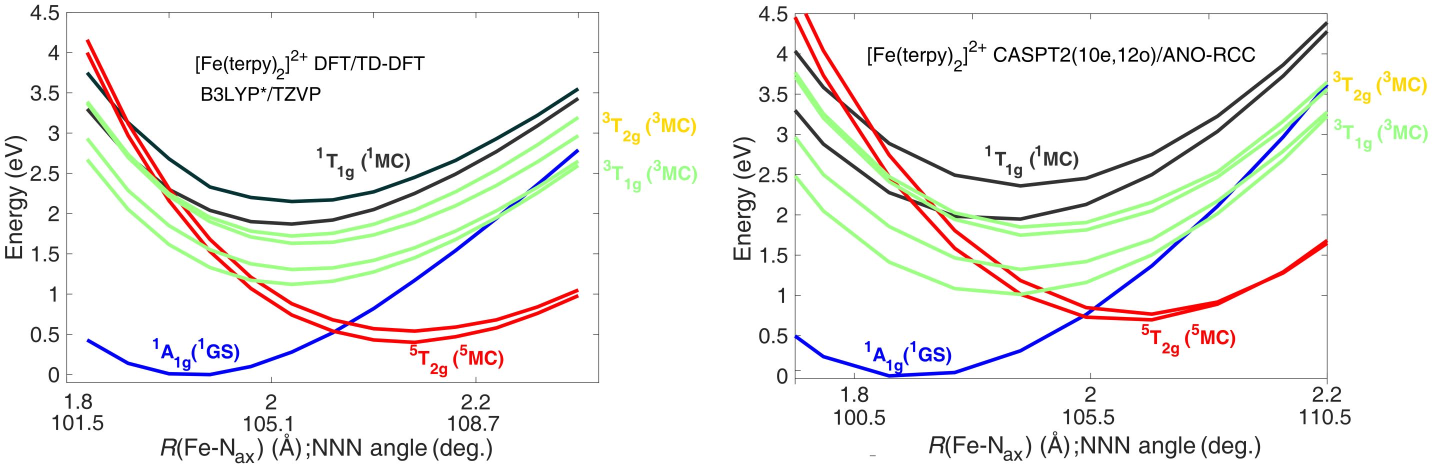

Supplementary Figure 8: Potential energy surfaces of $[Fe(terpy)_2]^{2+}$ along a combined coordinate (Fe-N_{ax} bond length, NNN angle) that connects the LS ($^1A_{1g}$) and HS ($^5T_{2g}$) minima, calculated by the DFT/TD-DFT approach used in the present work (RKS/TD-DFT singlets and triplets, UKS quintets, left) and CASPT2 (from Supplementary Ref. 6, right).

Furthermore, we evaluated the analogous comparison for the closely-related $[Fe(bipy)_3]^{2+}$ complex, for which PESs including those for MLCT states are available at the reference CASPT2 level.[7] Here, as can be seen in the below figure (Supplementary Figure 9), the same conclusions are reached as for $[Fe(terpy)_2]^{2+}$: the overall agreement is rather good (note that the smaller number of curves is due to the higher symmetry of $[Fe(bipy)_3]^{2+}$, the structure of $[Fe(terpy)_2]^{2+}$ significantly departs from octahedral symmetry by axial distortion).

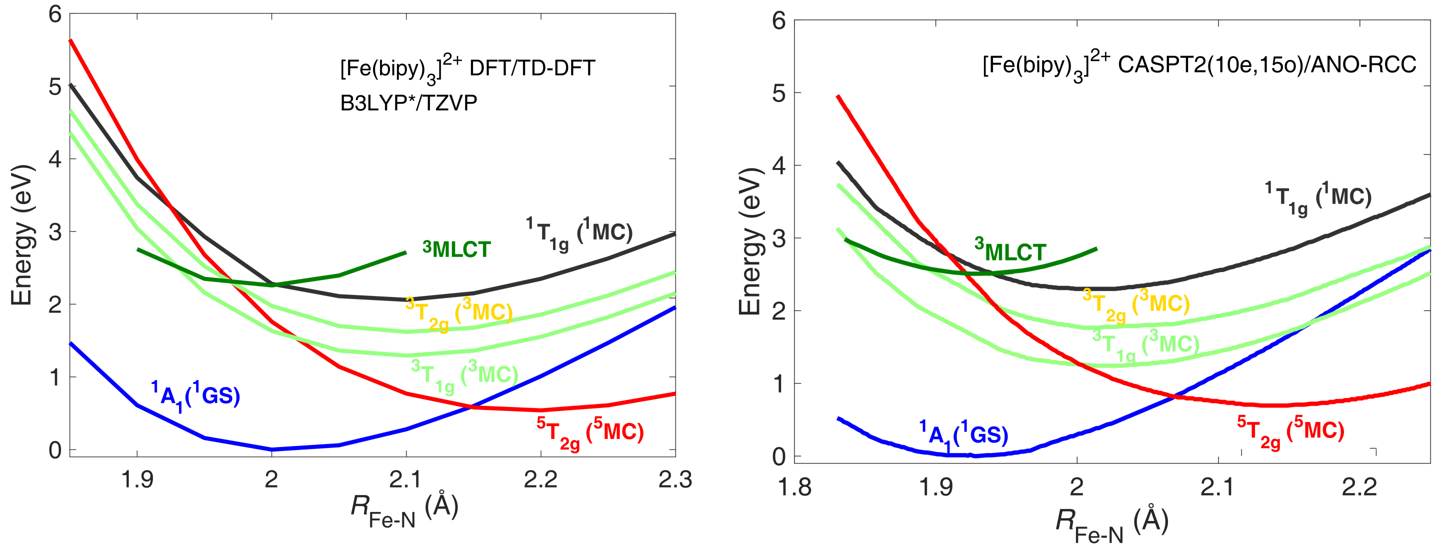

Supplementary Figure 9: Potential energy surfaces of $[Fe(bipy)_3]^{2+}$ along the symmetric Fe-N stretching coordinate, calculated by the DFT/TD-DFT approach used in the present work (RKS/TD-DFT singlets and triplets, UKS quintets, left) and CASPT2 (from Supplementary Ref. 7, right).

Finally, the third set of PESs shown in Supplementary Figure 10 demonstrates the close similarity of the excited-state energetics of $[Fe(terpy)_2]^{2+}$ and $[Fe(bipy)_3]^{2+}$; for both, we utilised the DFT/TD-DFT methodology used in the present work (RKS TD-DFT + UKS). This justifies the utilisation of the second set of PESs for $[Fe(bipy)_3]^{2+}$, which is though not exactly the same complex as $[Fe(terpy)_2]^{2+}$, but for benchmarking purposes the differences are clearly negligible.

Last, we present the result of our additional dynamics simulations, in which we shifted the energy of the quintet states by +0.2 eV, according to the CASPT2 energetics. We calculated 100 trajectories, the results are compared in the figure below (Supplementary Figure 11) to the population dynamics of our original simulation with unshifted energies. This figure demonstrates good overall agreement with the only notable difference in the timescale of the quintet population growth, which is faster for the new simulation, as expected from the reduction of the decisive triplet-quintet energy gaps around the FC geometry, caused by the +0.2 eV quintet energy shift. Importantly, all these results point in the same direction validating the accuracy of our DFT/TD-DFT methodology.

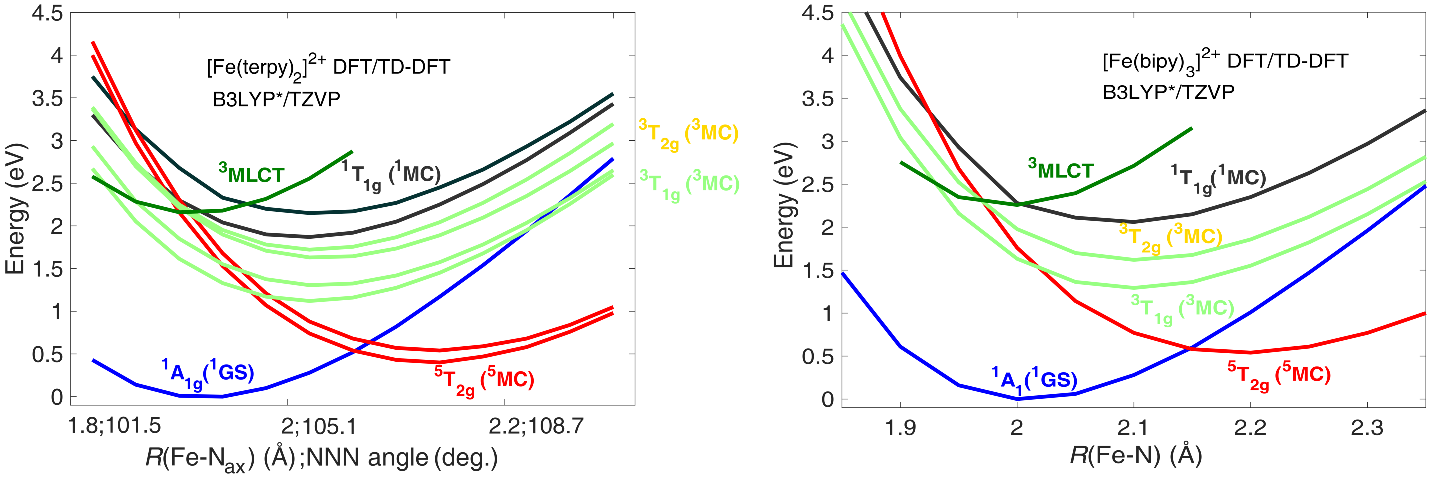

Supplementary Figure 10: Potential energy surfaces of $[Fe(terpy)_2]^{2+}$ (left) and $[Fe(bipy)_3]^{2+}$ (right), calculated by the DFT/TD-DFT approach used in the present work (RKS/TD-DFT singlets and triplets, UKS quintets, left).

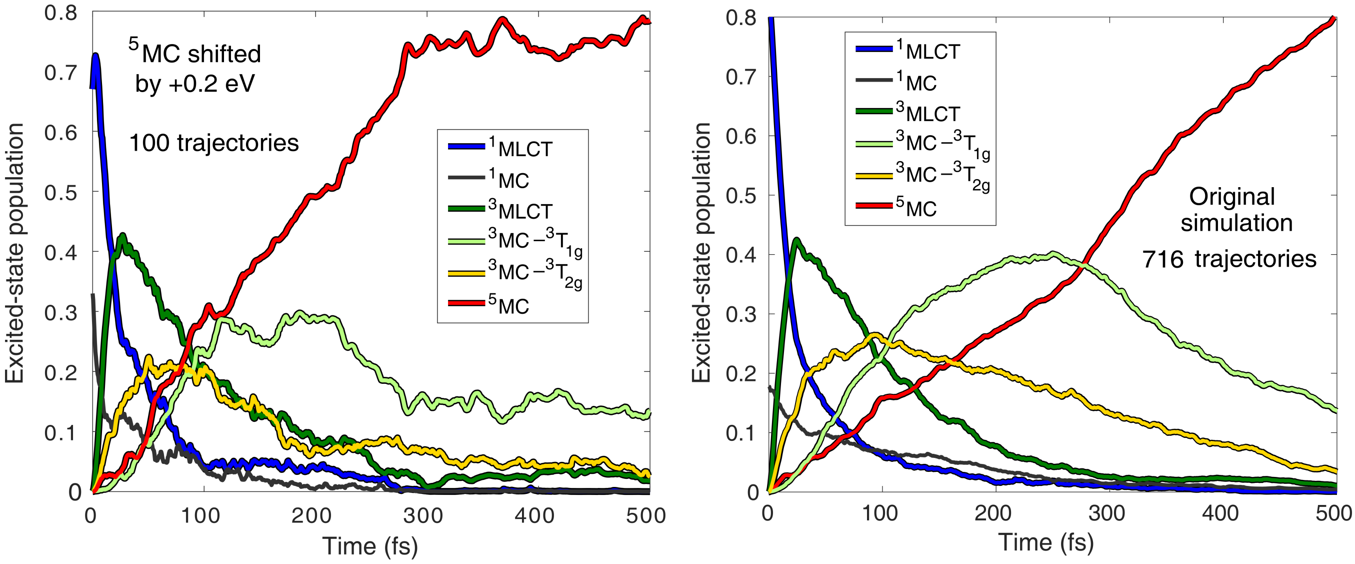

Supplementary Figure 11: Effect of +0.2 eV ^{5}MC energy shift on the simulated population dynamics (the energies of all other states were unchanged). The left panel shows the results applying the +0.2 eV ^{5}MC energy shift, while the right panel displays the original simulated population dynamics (identical to Figure 3 of the main article).

Supplementary References

[1] Köppel, H., Domcke, W. & Cederbaum, L. S. Multimode Molecular Dynamics Beyond the Born-Oppenheimer Approximation, 59–246 (John Wiley & Sons, Ltd, 1984).

[2] Plasser, F., Gómez, S., Menger, M. F. S. J., Mai, S. & González, L. Highly efficient surface hopping dynamics using a linear vibronic coupling model. Phys. Chem. Chem. Phys. **21**, 57–69 (2019).

[3] Zobel, J. P., Heindl, M., Plasser, F., Mai, S. & González, L. Surface hopping dynamics on vibronic coupling models. Acc. Chem. Res. **54**, 3760–3771 (2021).

[4] Pápai, M. Towards simulation of Fe(II) low-spin → high-spin photoswitching by synergistic spin-vibronic dynamics. J. Chem. Theory Comput. **18**, 1329–1339 (2022).

[5] Xie, W., Sapunar, M., Došlić, N., Sala, M. & Domcke, W. Assessing the performance of trajectory surface hopping methods: Ultrafast internal conversion in pyrazine. J. Chem. Phys. **150**, 154119 (2019).

[6] Pápai, M., Vankó, G., de Graaf, C. & Rozgonyi, T. Theoretical investigation of the electronic structure of Fe(II) complexes at spin-state transitions. J. Chem. Theory Comput. **9**, 509–519 (2013).

[7] Sousa, C. et al. Ultrafast deactivation mechanism of the excited singlet in the light-induced spin crossover of $[Fe(2,2'\text{-bipyridine})_3]^{2+}$. Chem. Eur. J. **19**, 17541–17551 (2013).
