# Supplementary material for: Branching mechanism of photoswitching in an Fe(II) polypyridyl complex explained by full singlet-triplet-quintet dynamics
Source: Commun Chem. 2023 Jan 9;6:7. doi: 10.1038/s42004-022-00796-z (PMC9829715; doi:10.1038/s42004-022-00796-z)
Supplement: Supplementary file 3 — Description of Additional Supplementary Files [file 42004_2022_796_MOESM3_ESM.docx]

Description of Additional Supplementary Files

**File name:** Supplementary Data 1

**Description:** Numerical parameters of the utilized model

**File name:** Supplementary Data 2

**Description:** Initial geometries, velocities (given in atomic units) and initial state indices corresponding to the adiabatic/spin-diabatic electronic basis
